# Progress in Medicine: Experts Take Stock

**DOI:** 10.1371/journal.pmed.1001933

**Published:** 2015-12-29

**Authors:** The PLOS Medicine Editors, Andrew Beck, Ewan Birney, Manuel Graeber, James Tumwine, Phillipa Hay, Hyeong Sik Ahn, Anushka Patel, Philipp du Cros, Lorenz von Seidlein, Nick Wareham, Nicola Low

**Affiliations:** 1 Public Library of Science, San Francisco, California, United States of America; 2 Department of Pathology, Harvard Medical School, Beth Israel Deaconess Medical Center, Boston, Massachusetts, United States of America; 3 Joint Director, European Bioinformatics Institute, Hinxton, United Kingdom; 4 Neuropathologist and Director, Brain Tumour Research, University of Sydney, Sydney, Australia; 5 Professor of Paediatrics and Child Health, School of Medicine, Makerere University, College of Health Sciences, Kampala, Uganda; 6 School of Medicine, University of Western Sydney, Sydney, Australia; 7 Department of Preventive Medicine, College of Medicine, Korea University, Seoul, Korea; 8 The George Institute for Global Health, University of Sydney, Sydney, Australia; 9 Head of the Manson Unit, Médecins Sans Frontières (MSF), London, United Kingdom; 10 Mahidol-Oxford Tropical Medicine Research Unit (MORU), Mahidol University, Bangkok, Thailand; 11 MRC Epidemiology Unit, University of Cambridge, Cambridge, United Kingdom; 12 Institute of Social and Preventive Medicine, University of Bern, Bern, Switzerland

## Abstract

In the year-end editorial, the *PLOS Medicine* editors ask 11 researchers and clinicians about the most relevant challenges, promising research, and important initiatives in their fields as we head into 2016.

## Introduction

For the 2015 end-of-the-year editorial, *PLOS Medicine* asked 11 researchers and clinicians spanning a range of specialties to comment on the state of their field and what they expect or hope to see next year. From cardiovascular diseases and diabetes to cancer to infectious diseases, from new research and technologies to clinical practice, and from training to health policy and strategy, our contributors had plenty to say. Here’s to a healthy 2016!

### Andrew Beck, Department of Pathology, Harvard Medical School, Beth Israel Deaconess Medical Center

#### What was the biggest “game changing” paper in the cancer biology field in the last year?

An important paper from 2015 is the study by Joann Elmore and colleagues regarding diagnostic discordance in the pathologic assessment of preinvasive breast neoplasia [1]. In this study, the authors show very high levels of diagnostic agreement in the diagnosis of invasive breast cancer (96%) but significantly lower levels of agreement for the pathological assessment of preinvasive breast lesions, including a rate of only 48% agreement for the diagnosis of benign breast lesions with atypia. Given the widespread implementation of screening mammography and the continuing development of imaging technologies to detect breast neoplasia at increasingly early stages, the pathological assessment of early breast lesions is becoming increasingly common. The pathological assessment of breast biopsies plays a critical role in the management of breast abnormalities identified by radiological imaging and may be the single factor determining a therapeutic recommendation of routine screening versus surgery, chemotherapy, and radiation therapy. The study by Elmore and colleagues points to a major current problem area in diagnostic breast pathology, and this work demonstrates that the development of innovative new approaches—potentially leveraging morphological and molecular data and new developments in machine learning and artificial intelligence—could have significant impact by assisting breast pathologists in making accurate, reproducible, and informative diagnoses for this challenging area of breast pathology.

### Ewan Birney, Joint Director, European Bioinformatics Institute

#### What's next in the clinical genomics field, and will personalized genomic data ever be freely available?

The biggest transformation in my view has been in rare diseases, with the flagship Deciphering Developmental Disorders project in the United Kingdom being an excellent example of how good, systematic genomic data (in this case whole exome) can both transform the diagnosis for children and enable new discoveries. With other projects, such as ClinGen in America and the 100K Genomes in the UK, this will impact more and more people over the next year.

I am very excited about the impact of real-time sequencing and longer reads in genomics. The work by Nick Loman on Ebola in West Africa and by Justin O’Grady on urinary tract infections and sepsis is really showing the power of handheld sequencing. New technologies are going to bring down the cost for long reads in genomics, and I think this will have an impact across genomics, first in structural variation but then more broadly.

Genomics will always cost something—less and less for the actual sequencing, but the sample management and informatics also have to be factored in. Therefore, the question about personalized genomic data is not about whether these data will be freely available but rather how they will be integrated into the health care systems around the world. A number of countries—the UK (100K Genomes, GoShare in Scotland), the US (Precision Medicine Initiative [PMI, pdf]), Denmark (BioBank), Estonia, and Finland—have quite well-developed plans to leverage genomic data more broadly, often with a strong health care component. Noninvasive prenatal testing has become a strong use case for genomic data. Infection diagnosis and control is being transformed. Thus, genomics is a very broad-based set of technologies that will have a lasting impact on the future.

### Manuel Graeber, Neuropathologist and Director, Brain Tumour Research, University of Sydney

#### Over a century has passed since the discovery of Alzheimer disease and the description of Lewy bodies associated with Parkinson disease, but we still don’t have effective treatments to slow these and other neurodegenerative diseases. What are the hurdles? Has the field become sidetracked?

Neuroinflammation has become a hugely popular term, but the word's meaning has also changed. It was once used to refer to immune-driven pathology in the nervous system, such as multiple sclerosis, but is now effectively equated with the increased expression of certain cytokines or other markers of activation by the brain's resident immune cells, the microglia. The problem is that these cells become activated in most if not all brain diseases and even under subclinical conditions, i.e., their observed response is highly sensitive but also very unspecific. The cells can even fall victim to disease themselves.

Importantly, the apparent main function of the microglia in normal brain, their role in the maintenance of synapses in the central nervous system (CNS), has become overshadowed by the contemporary focus on microglia as inflammatory cells. Worse than that, based on semantics alone, patients may be treated with anti-inflammatory drugs.

It is particularly attractive for drug companies to find new uses for existing drugs, as no additional research and development (R&D) is required. However, it remains to be shown that these drugs provide any benefit for patients with conditions such as dementia, depression, or schizophrenia, as clinical trials have failed. Chronic pain, obesity, and autism have also been considered as brain inflammatory conditions at one time or another, but with no clear implications for treatment.

### James Tumwine, Professor of Paediatrics and Child Health, School of Medicine, Makerere University, College of Health Sciences, Uganda

#### What are the biggest challenges to training to be a medic in a resource-limited setting, and what single change would make the biggest difference to health care professional training and patient outcomes in the future?

It is tough training to be a medic in countries where resources are scarce. Challenges abound, but most serious of all are the inequities in health care. Getting into medical school is nearly impossible for students from low-income families, who are in the majority in countries such as Uganda, and finding some income support during this training is a nightmare. What is really most frustrating is having to deal with very ill patients afflicted, largely, by infectious diseases, while also handling a resurgence of noncommunicable disease singly or in combination. At the same time, resources for care are extremely limited, with medicines and reagents for tests often chronically out of stock. What is rewarding, however, is that one learns to handle patients using limited resources, utilizing the skills of history taking and clinical evaluation. What would make the biggest difference, in my view, is more investment into preventive programs and provision of free health care at the point of delivery and not sending health care to the market place.

### Phillipa Hay, University of Western Sydney

#### What are the greatest barriers to recognizing mental health conditions and accessing mental health care in 2015, and what can be done to overcome these barriers in 2016?

Mental health disorders are now “leaders” in the global burden of disease [2]. However, there continue to be wide gaps in equable distribution of access to evidence-based health care and health care providers. This is not merely a matter of a country’s net wealth. In some high-income countries, there is an excess of professionals such as psychiatrists, but even then maldistribution leaves many people with inadequate treatment and support and a significantly reduced life expectancy. Why is this so?

Amongst the numerous barriers to identification of mental health disorders, stigma stands out as a pernicious and persistent force [3]. Stigma promotes mental health disorders being hidden and going “underground.” Overt and covert actions that engender a fear of discrimination, such as questions on visa application forms about a mental illness history, contribute. Stigma also influences attitudes of health professionals and care providers to avoid people with psychiatric illness, with resulting suboptimal physical health care.

Improved training of health professionals more broadly, including in stigma awareness, is needed to reduce barriers. Public health campaigns and interventions such as “mental health first aid” have had some success in reducing stigma and improving mental health literacy in the wider community [4]. Further trials need to test the effectiveness of the translation across geographic and linguistic boundaries and broader dissemination for such evidence-based community approaches.

### Hyeong Sik Ahn, Department of Preventive Medicine, College of Medicine, Korea University

#### What can be learned from the expansion of cancer screening and treatment in Korea and the subsequent efforts to curb cancer overdiagnosis and overtreatment?

Following the introduction of a government-initiated cancer screening program in 1999, cancer screening and treatment has become a big industry in Korea. One aspect of this program is a paid fee program. Under this “fee-for-service” system, many hospitals market general health screening programs that may include high technology-oriented examinations such as magnetic resonance imaging (MRI) or positron emission tomography (PET) scans. Many general practitioners also offer cancer screening services using their own ultrasonography or endoscopy machines in their offices. For many of the patients diagnosed with cancer, it is often the case that part of the treatment regimen includes surgery. During the past decade, hospitals have expanded screening facilities and hired more surgeons and radiographers. This has fueled a screening and cancer surgery industry. Early detection and treatment may cause increased incidence of cancer, which may induce needless treatment (often surgery) for tumors growing too slowly to do any harm [5]. In a profit-oriented and incentive-loaded health care system, it is hard for individual clinicians to provide the best care for their patients while at the same time complying with government-backed, evidence-based cancer screening guidelines.

The virtue of early detection and screening is appealing and convincing, and it is hard to persuade the general public and clinicians to see a negative flip side. However, society should be aware that active health services can lead people to unnecessary treatments, and these may not necessarily improve lives. Worse still, they can produce complications or even earlier death because of unnecessary medical procedures. The Physician Coalition for Prevention of Overdiagnosis of Thyroid Cancer managed to achieve a victory in this regard: after sending an open letter to the public in 2014, there was a 35% reduction in thyroid cancer surgeries during one year [6]. This example reveals how a small number of physicians can influence and even change the direction of medical care by raising health care issues to the wider public. This learning and cultural shift is essential for dealing with the issue of overdiagnosis, and it also facilitates the transition of health services research into the implementation of clinical practice. Looking ahead, the challenge now is to influence similar changes in other settings and medical fields.

### Anushka Patel, The George Institute for Global Health, University of Sydney

#### Low- and middle-income countries bear a growing burden of noncommunicable diseases, in particular vascular disease. Which recent or upcoming research developments make the most meaningful progress toward improving this aspect of long-term health in these settings?

The latest data from the Global Burden of Disease study, published in 2015 and reflecting the status in 2013, confirm that noncommunicable diseases prominently feature in the leading causes of life years lost globally, and ischemic heart disease ranked at the top of this list [7]. Important research published in 2015 highlighted one of the major concerns regarding the control of cardiometabolic conditions in low- and middle-income countries, where 80% of cardiovascular deaths occur [8]. The Prospective Urban Rural Epidemiology (PURE) study published data on the availability of four “essential medications” for cardiovascular disease prevention among individuals with established disease (secondary prevention). There was limited availability of these medications in many low- and middle-income countries, especially in rural communities. Furthermore, even if these medications were available, they were found to be potentially unaffordable for up to 60% of individuals in some low-middle- and low-income countries. Such a crisis is not unprecedented—in the face of the HIV/AIDS pandemic, a global multisectoral response addressed, among a range of other outcomes, the availability and affordability of antiretroviral drugs. Whether this can serve as a model for the cardiometabolic pandemic is yet to be seen.

### Philipp du Cros, Head of the Manson Unit, Médecins Sans Frontières (MSF), London, UK

#### What is the single biggest change we could make to improve tuberculosis (TB) treatment and response in 2016?

Multidrug-resistant tuberculosis (MDR-TB) is a public health emergency that threatens progress in TB care and control. An estimated 480,000 cases of MDR-TB occurred in 2014 [9]. Standard MDR-TB treatment is hugely inadequate: it is lengthy (20–24 months, with 8 months of injected drugs), toxic, expensive, and has a 50% failure rate. In addition, treatment of more resistant strains has even worse outcomes. While two new drugs—bedaquiline and delamanid—offer hope, access to them currently remains limited, with many patients who meet the criteria in global guidelines for treatment with these drugs unable to start regimens. In 2016, a more urgent scale-up of these drugs is required. Finally, in the longer term, clinical trials are needed to define the best drug combinations enabling shorter, more effective regimens.

### Lorenz von Seidlein, Mahidol-Oxford Tropical Medicine Research Unit (MORU), Mahidol University, Bangkok, Thailand

#### What’s the next big hurdle in the fight to eliminate malaria, and what can be done in 2016 to get past it?

Increased attention and funding since the beginning of the century has reduced the current burden of malaria. This progress has been attributed to the introduction of insecticide-treated bed nets and the inclusion of artemisinin combination therapy in case management [10]. However, increasing resistance of insect vectors to insecticides and *Plasmodium falciparum* to artemisinins threaten these gains. Where there used to be pockets of insecticide resistance, there are now only pockets of susceptibility left [11]. Artemisinin-resistant *P*. *falciparum* strains have replaced the susceptible wild type in many parts of Cambodia [12], one of the sites where chloroquine, pyrimethamine, and sulphadoxine resistance originated [13] and where malaria incidence is again on the rise. These resistant *P*. *falciparum* strains have spread across Southeast Asia and are now encroaching on the subcontinent, the traditional gateway of malaria resistance to Africa, where large populations are at risk for resurgent falciparum malaria [14]. Meanwhile, the public health response to vector and parasite resistance remains firmly rooted in the last century. In the absence of a quick and appropriate adjustment of control strategies to the ever-changing reality of malaria epidemiology, resurgence is a real possibility in places that have managed to control the disease over the last decade.

### Nick Wareham, MRC Epidemiology Unit, University of Cambridge, UK

#### Diabetes is at epidemic levels. How can its progress be halted?

As the question implies, the prevalence of diabetes has increased markedly across all continents. Part of that increase is undoubtedly due to an increase in the incidence of diabetes, but much is attributable to the ageing of populations (since type 2 diabetes is such an age-dependent disease), to improved detection (reducing the prevalence of undiagnosed disease), and to improved survival of people with diabetes. Our immediate public health goal should be to limit and ideally reverse the increase in the incidence of the condition, rather than to impact on the overall prevalence since this is likely to continue to rise in the future. Individual-level lifestyle interventions in people known to be at high risk, such as those with prediabetes, can reduce risk of progression by 58% in randomized controlled trials [15]. The effectiveness in real world settings is likely to be less. Although such intervention programs are effective for those individuals who participate in them, the impact of these high-risk prevention approaches on the epidemic of diabetes is relatively small. Much greater public health benefit can be achieved by population approaches that seek to promote small changes in behavior in large groups of people at low to moderate risk. Although such changes make little impact on individual-level risk, when they are amassed across large populations, this strategy makes the greatest impact on the epidemic of diabetes. There is no single intervention that can effect such population-level changes; instead, a wide range of different approaches are needed. Perhaps the biggest initial step is for governments to recognize and accept that diabetes is a clinical manifestation of a societal problem that requires societal solutions.

### Nicola Low, Institute of Social and Preventive Medicine, University of Bern

#### What’s the most important challenge in treating/preventing HIV and other sexually transmitted infections that will need to be resolved in the next year?

When the World Health Organization (WHO) announced in July 2015 that Cuba was the first country to have received validation for the elimination of mother-to-child transmission of HIV and syphilis [16], the world’s media only mentioned HIV, even though syphilis in pregnancy causes more fetal and perinatal infections [17] than HIV [18] and prevention of congenital syphilis costs a fraction of the price of preventing HIV transmission [19]. Those in the field of “other sexually transmitted infections” (STIs) urgently need to quantify accurately the years of life lost and years lost due to disability to STI to show the gains of investing in prevention and control and reverse falls in funding. The unrecognized burden of STI includes conditions such as preterm births, low birth weight and perinatal deaths caused by STIs in pregnancy, cervical cancer caused by human papillomavirus infections, and antimicrobial resistance. For HIV infection, WHO guidelines in 2015 recommended immediate antiretroviral therapy for anyone with HIV and pre-exposure prophylaxis for HIV-negative people at high risk of becoming infected [20]. The major challenge must be to determine whether enough people in the most heavily affected countries can be encouraged to find out their HIV status before embarking on testing and treating our way out of the HIV epidemic.

## Abbreviations

CNS: central nervous system
MDR-TB: multidrug-resistant tuberculosis
MORU: Mahidol-Oxford Tropical Medicine Research Unit
MRI: magnetic resonance imaging
MSF: Médecins Sans Frontières
PET: positron emission tomography
PMI: Precision Medicine Initiative
PURE: Prospective Urban Rural Epidemiology
R&D: research and development
STI: sexually transmitted infection
TB: tuberculosis
WHO: World Health Organization
